# Rethinking Digital Outcome Measures for Amyotrophic Lateral Sclerosis Trials: Consensus Priorities for Actigraphy Harmonization, Validation, and Regulatory Readiness

**DOI:** 10.2196/106677

**Published:** 2026-11-12

**Authors:** Frederik Steyn, Rakesh Pilkar, Nadia Sethi, Lori Heller, Cory Holdom, Ruben P A van Eijk, Marcin Straczkiewicz, Katherine M Burke, James D Berry, Sanjay Chandriani, Gordon Jewett, Emma Hodson-Tole, Collin Hovinga, Fernando Vieira, Christine Guo

**Affiliations:** 1 UQ Centre for MND Research, School of Biomedical Sciences, The University of Queensland, Research Road, St Lucia, Brisbane, Queensland, 4072, Australia, 61 0414653551; 2 Ametris, Pensacola, FL, United States; 3 North Star ALS, Santa Barbara, CA, United States; 4 Nuffield Department of Clinical Neurosciences, University of Oxford, Oxford, England, United Kingdom; 5 Department of Neurology & Biostatistics, University Medical Center Utrecht, Utrecht, The Netherlands; 6 AGH University of Krakow, Krakow, Poland; 7 Healey & AMG Center for ALS, Massachusetts General Hospital, Boston, MA, United States; 8 Trace Neuroscience, South San Francisco, CA, United States; 9 Department of Clinical Neurosciences, University of Calgary, Calgary, AB, Canada; 10 Department of Life Science, Manchester Metropolitan University, Manchester, England, United Kingdom; 11 Critical Path Institute, Tucson, AZ, United States; 12 ALS Therapy Development Institute, Watertown, MA, United States

**Keywords:** digital clinical outcome assessments, actigraphy, accelerometry, regulatory pathways, patient-centered outcomes, amyotrophic lateral sclerosis, clinical trial design

## Abstract

Amyotrophic lateral sclerosis (ALS) clinical trials need outcome measures that complement established end points while capturing how people function in daily life. In this viewpoint, we draw on our collective experience in ALS research, clinical trials, digital-measure development, and regulatory science, together with published evidence, a preliminary patient survey, stakeholder discussions, and experience from multisite actigraphy studies. We argue that the field now has a substantial base of longitudinal data and candidate measures, but lacks the coordinated framework needed to compare, prioritize, and validate them across studies. We identify 4 linked priorities: adopt fit-for-purpose measures in stages and within defined contexts of use; harmonize data collection and analysis; use data to shortlist and validate measures across cohorts; and prepare actigraphy methods for trial adoption through early industry and regulatory engagement. Across these priorities, actigraphy should complement rather than replace established clinical outcomes, with its integration guided by patient relevance, ALS phenotypic heterogeneity, participant, and caregiver burden, and the intended context of use. Our central message is that progress now depends less on generating additional isolated datasets or measures than on coordinating the evaluation of existing assets, and aligning future evidence generation.

## Background and Consensus Development

### Amyotrophic Lateral Sclerosis: A Complex Therapeutic Landscape

Amyotrophic lateral sclerosis (ALS) is a fatal neurodegenerative disorder characterized by progressive upper and lower motor neuron degeneration, resulting in muscle weakness, spasticity, paralysis, and respiratory failure [1]. Although ALS is clinically defined by progressive motor neuron degeneration, it is now understood as part of a broader neurodegenerative spectrum in which cognitive and behavioral changes occur in up to half of individuals, with a smaller subgroup meeting criteria for frontotemporal dementia [2,3]. Variability in site of onset, motor phenotype, progression rate, and nonmotor involvement together produces substantial clinical heterogeneity, complicating therapeutic development, trial design, and outcome measurement. Although many ALS trials have not met their primary end points, therapeutic development is advancing, with riluzole, edaravone, and tofersen currently approved disease-modifying options [4,5]. While current treatments provide modest benefit or apply to specific patient subgroups, these advances highlight continued progress in ALS therapeutics and the expanding need to evaluate emerging therapies effectively.

### Outcome Measurement Must Keep Pace With ALS Therapeutic Development

As ALS therapeutic development advances, trials require outcome measures that demonstrate clinically meaningful benefit in how people living with ALS feel, function, and survive [6]. Trial failure remains multifactorial, reflecting disease heterogeneity, target selection, patient stratification, trial design, and end point sensitivity [7]. No single measure can resolve these barriers, but improving outcome measurement remains a tractable priority for evaluating therapies efficiently and interpreting treatment effects with confidence.

ALS studies primarily rely on survival and daily functioning–based end points such as the Amyotrophic Lateral Sclerosis Functional Rating Scale–Revised (ALSFRS-R), with more than 80% of pivotal studies using the ALSFRS-R total score [4,8]. The ALSFRS-R is simple and widely applicable [8,9], but it is also multidimensional, subjective, and variably administered [10]; therefore, changes in total score may obscure domain-specific patterns of functional decline [8,9]. Alongside clinical end points, biological markers are needed to assess target engagement, pharmacodynamic response, and disease activity. Neurofilament light chain has strengthened the assessment of neuronal injury and treatment response in ALS [11], but it does not directly capture daily function. Together, biological markers and sensitive functional outcome measures may provide a more complete framework for evaluating biological activity and meaningful patient benefit.

Wearable digital health technologies (DHTs), particularly actigraphy, offer complementary tools to strengthen ALS drug development by enabling repeated, remote, and objective assessment of movement-related function in real-world settings [6,12]. Unlike episodic clinical assessments, actigraphy can generate high-resolution data on functional change between visits, with potential to detect subtle changes in mobility, limb activity, activity intensity, and daily routines. These measures may improve trial interpretation, support earlier decision-making, and reduce reliance on sparse clinic-based assessments, provided they are implemented with acceptable participant burden. To implement actigraphy clinically, the field must establish analytical validity and clinical meaningfulness, demonstrate patient and regulatory acceptability, and develop harmonized protocols.

Evidence supporting the use of actigraphy in ALS has advanced beyond initial proof of concept [9,12]. Longitudinal studies have shown that passively collected movement measures can characterize functional differences, correlate with established clinical outcomes, and detect change over time; selected measures have also been associated with survival [13-18]. More recent work has begun to address specific validation gaps, including evaluation of free-living upper-limb measures in larger cohorts and ALS-specific analytical and clinical validation of ankle-derived step counts [17,19]. Preliminary studies also suggest that wearable monitoring is generally acceptable to people living with ALS, although technical difficulties, incomplete data, and individual differences in burden remain relevant [20]. Collectively, these studies provide a substantial foundation of longitudinal data and candidate measures with which to evaluate ALS-related functional change.

However, largely independent studies have generated these data and measures using different devices, sensor locations, sampling frequencies, wear schedules, definitions of valid wear, preprocessing methods, and derived features [9]. This fragmentation makes candidate measures difficult to compare directly, to reproduce across cohorts, and to develop into a cumulative evidence base. External validation across independent cohorts and evidence of responsiveness to treatment consequently remain limited, and it is not yet clear which measures are sufficiently reliable, interpretable, patient-centered, and sensitive for specific clinical-trial roles [9,12]. Therefore, the principal barrier is no longer whether actigraphy contains a disease-related signal or whether further data and measures can be generated, but rather the lack of a coordinated, field-wide approach for evaluating and building on what already exists. The field, therefore, needs a shared, stakeholder-informed framework to apply common analytical approaches to existing datasets where feasible, align prospective data-collection protocols, prioritize candidate measures for cross-cohort validation, incorporate patient and caregiver requirements, and define a staged pathway toward trial adoption and regulatory engagement [6,9,12].

We aim to draw on current evidence, preliminary patient input, stakeholder expertise, and multisite experience to articulate four linked priorities for advancing actigraphy in ALS trials: (1) staged adoption of fit-for-purpose measures across defined contexts of use, (2) harmonization of data collection and analysis, (3) data-driven shortlisting and cross-cohort validation, and (4) adoption-ready trial implementation supported by early industry and regulatory engagement. Through these priorities, we propose a practical roadmap for converting existing actigraphy data and candidate measures into a coordinated evidence base for complementary use alongside established clinical outcomes.

### Stakeholder and Multisite Inputs Informing This Viewpoint

Priorities summarized in this viewpoint were informed by a multidisciplinary workshop held at the ActiGraph Digital Data Summit 2025 in Pensacola, Florida, followed by a targeted ActiGraph Digital Data Summit 2026 meeting in Atlanta, Georgia, focused on protocol refinement and implementation. These activities brought together people with ALS, caregivers, academic researchers, clinicians, biostatisticians, industry researchers, DHT developers, device manufacturers, regulatory experts, and stakeholders involved in clinical trials and end-point development.

Discussions focused on limitations of existing ALS clinical outcome measures, the potential role of actigraphy as a digitally derived clinical outcome measure, regulatory pathways for its use in ALS trials, and practical implementation. Inputs from patient survey findings, investigator-reported summaries of ongoing and planned actigraphy studies, and cross-site protocol comparison were interpreted alongside relevant literature to identify protocol heterogeneity, areas of convergence, and priorities for harmonization, validation, and staged adoption.

## Shared Priorities for Advancing Actigraphy in ALS

### Participant Input Supports the Feasibility of Actigraphy in ALS Trials

Participant input in digital outcome development was highlighted by an institutional review board–exempt survey on actigraphy in ALS. The study protocol (24-423-2011) was submitted to the BRANY SBER institutional review board (IRB00010793), which determined the survey to be exempt under 45 CFR 46.104(d)(2)(i) [21]. A total of 76 US adults with confirmed ALS were recruited through online patient, education, advocacy, and ALS community networks; provided informed consent; and completed the survey (Table 1), with an overall completion rate of 84%, although item-level response numbers varied.

**Table 1.** Demographic characteristics of survey participants (N=76).

Characteristic	Participants
Age (y), n (%)	
75 or older	2 (2.6)
65-74	19 (25.0)
55-64	23 (30.3)
45-54	11 (14.5)
35-44	15 (19.7)
25-34	6 (7.9)
Sex, n (%)	
Female	37 (49.3)
Time since symptom onset (y), n	
>5	29
3‐5	16
1‐3	24
<1	6
Participated in a clinical trial (% of respondents), n (%)	32 (41.7)

Participants had varied familiarity with clinical trials (32/76, 41.7% reported previous participation) and with actigraphy (34/76, 45% reported no prior familiarity and 21/76, 27% reported previous use; Figure 1A). Despite this variability, willingness to incorporate actigraphy into daily life was high. All respondents were open to wearing one or more devices; 76% (58/76) were willing to wear a device all day, and 32% (24/76) were willing to wear 3 or more devices simultaneously. Most were open to active and passive data collection, and interest increased when actigraphy could reduce in-person visits for care and/or trials (Figure 1B). Many respondents indicated that real-time data could help guide care decisions (63/76, 83%) and potentially improve quality of life (59/76, 77%). Comfort remained important, with 48% identifying it as a concern, although 36% reported no wearability concerns (Figure 1C).

**Figure 1.**
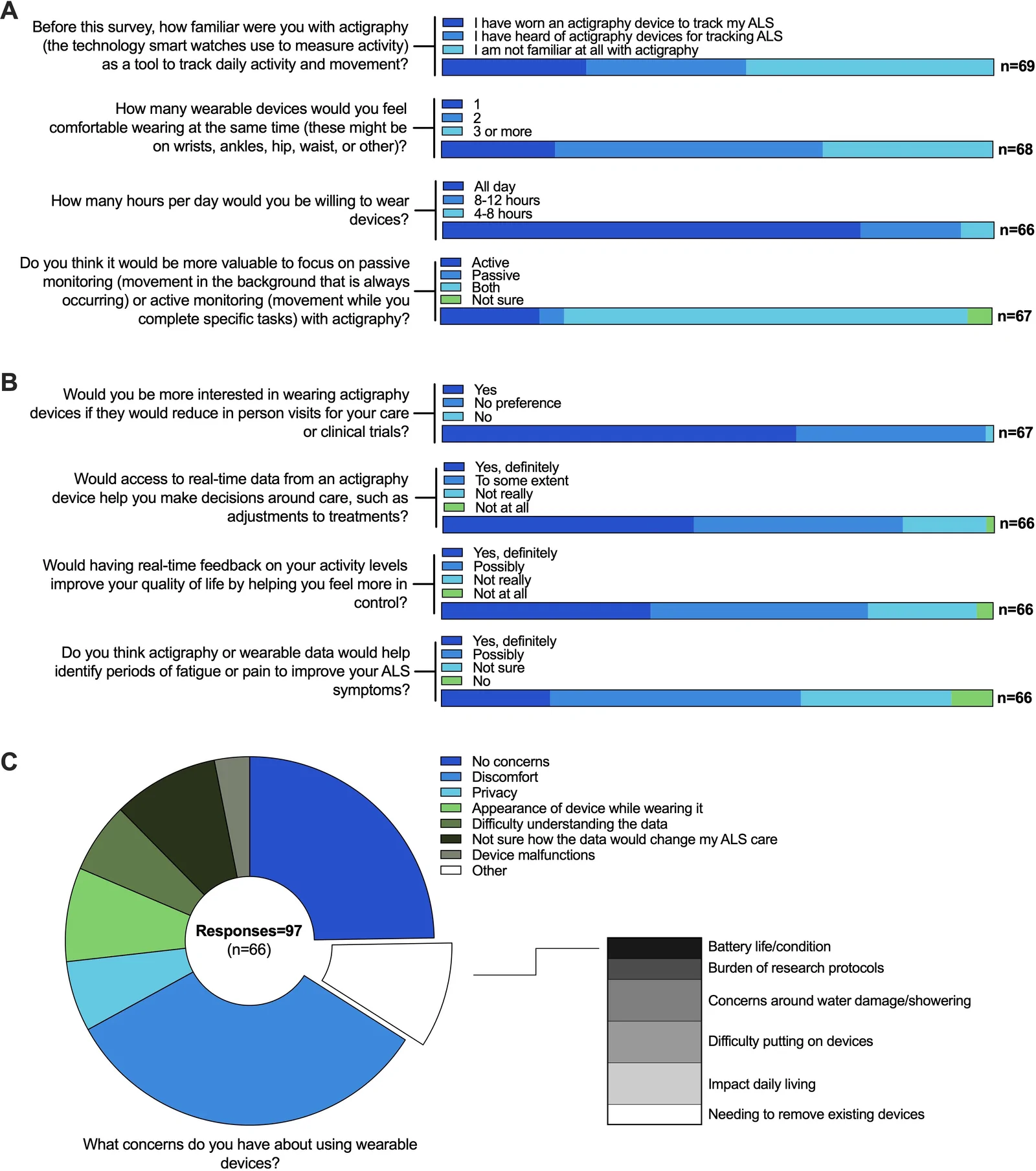
Survey responses on (A) familiarity with and prior use of actigraphy in amyotrophic lateral sclerosis (ALS), (B) perceived use in clinical care and trials, and (C) concerns regarding the use of actigraphy in people living with ALS. n: number of respondents. In panel C, respondents could select all concerns that applied; 66 respondents made 97 selections.

Most respondents saw value in actigraphy for improving the understanding of disease progression in care or trials (66/68, 97%), informing care decisions (66/66, 94%), and providing insight into daily-life impacts of ALS (63/69, 93%; Figure 2A). Respondents also endorsed the face validity of actigraphy for assessing functional change, particularly walking (66/67, 99%), hand and arm movement (63/67, 94%), sleep patterns (57/67, 85%), and activities of daily living (51/67, 76%; Figure 2B). These findings provide preliminary support for feasibility and acceptability from a participant perspective and align with prior multicenter survey findings on home-based digital monitoring in motor neuron diseases [22]. As an initial cross-section of patient perspectives, the findings may not represent the full diversity of the ALS community. Further participant and caregiver input will be needed as specific contexts of use are defined, particularly to establish relevance and feasibility across more diverse populations, disease stages, motor phenotypes, progression rates, and trial settings; the relatively high proportion of respondents living more than 5 years since symptom onset may also limit generalizability to people earlier in the disease course.

**Figure 2.**
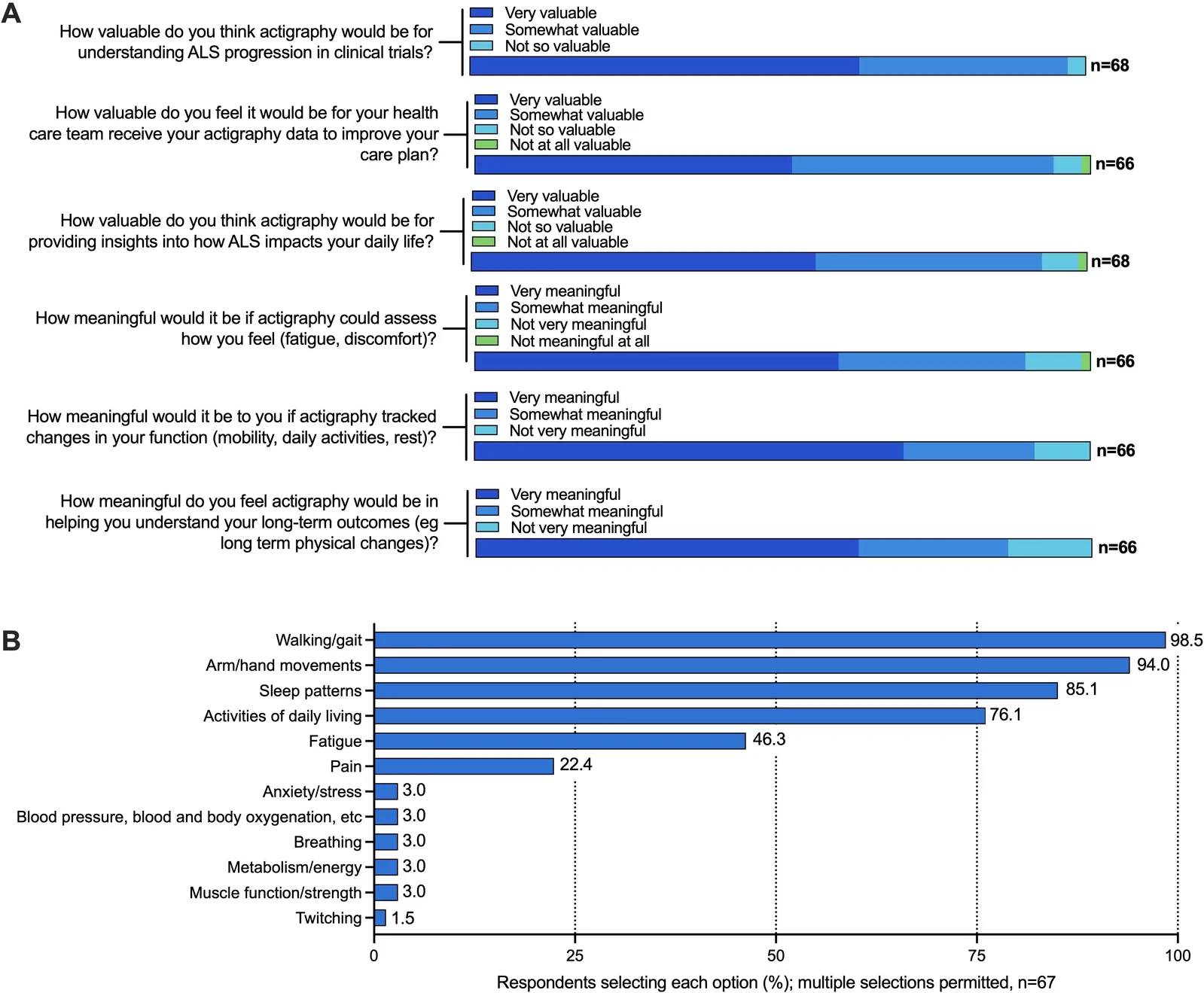
Survey responses on (A) the perceived value of actigraphy for monitoring amyotrophic lateral sclerosis (ALS) progression, informing care, and understanding daily-life impacts, and (B) participant perceptions of where actigraphy may best capture functional change and disease impact in ALS. For panel B, respondents could select more than one domain; percentages indicate the proportion of respondents selecting each option (n=67 respondents).

### Current End Point Limitations Define the Need for Supplemental Measures

#### The ALSFRS-R Remains the Dominant End Point Despite Persistent Limitations

Workshop discussions identified limitations of existing ALS end points as a key driver for the development of actigraphy-derived measures. Current ALS trials primarily rely on end points based on survival and daily functioning, such as the ALSFRS-R [4,5,7,8]. These end points provide direct evidence of survival and functional change, but detecting treatment effects may require longer follow-up and larger sample sizes, with interpretation complicated by heterogeneous progression, supportive interventions, and participant goals of care [6,7]. These constraints are particularly relevant in early-phase trials, where smaller, shorter studies may be insufficient to generate robust evidence for development decisions using established end points alone. Nevertheless, ALSFRS-R remains the most widely used functional end point in ALS efficacy trials [23,24]; it is simple, broadly applicable, clinically familiar, feasible for in-person or remote administration, and it captures function across multiple disease domains and can be related to the clinical disease stage [8]. However, this same multidomain structure creates interpretive challenges in heterogeneous ALS. Similar ALSFRS-R total scores may reflect different patterns of impairment across limb, bulbar, and respiratory domains (Figure 3), complicating interpretation when a therapy is expected to influence specific functional domains or patterns of functional change [8,23]. These limitations highlight the need for complementary measures that assess specific aspects of function more directly and repeatedly. Recent trial experience reinforces this need: tofersen showed biological effects without meeting clinical end points within the primary analysis period [25], whereas AMX0035 showed an ALSFRS-R signal in the phase 2 CENTAUR study [26] that was not replicated in the phase 3 PHOENIX study [27], highlighting how complementary objective measures may help interpret functional change and treatment signals.

**Figure 3.**
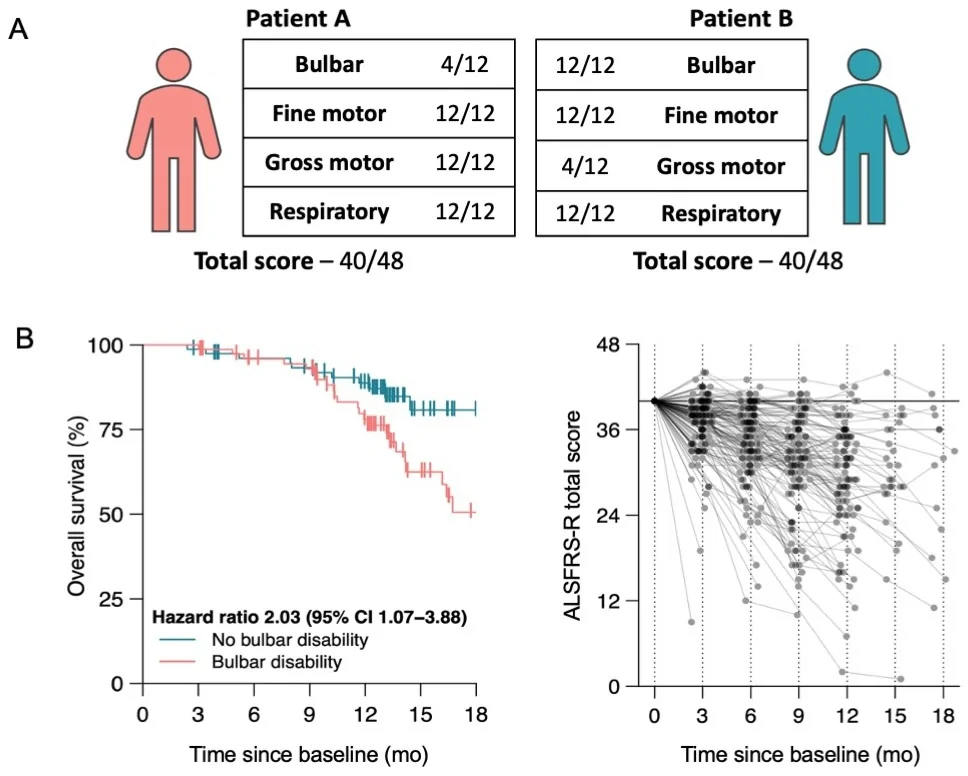
Interpretation challenges of the Amyotrophic Lateral Sclerosis Functional Rating Scale–Revised (ALSFRS-R) total score. (A) Representative participants with amyotrophic lateral sclerosis (ALS) with identical ALSFRS-R total scores but different functional profiles. All were selected from PRO-ACT (Pooled Resource Open-Access ALS Clinical Trials) and had a baseline total score of 40. (B) Survival trajectories of participants with ALS with and without bulbar disability, defined by an ALSFRS-R bulbar subdomain score ≤11. (C) Longitudinal ALSFRS-R total score trajectories from baseline.

A recurring view was that actigraphy-derived measures should supplement, rather than replace, ALSFRS-R scores or survival. Actigraphy-derived measures may address gaps not easily captured by episodic global assessments by providing objective, repeated assessment of movement-related function outside the clinic [6,9,12]. Depending on device placement, analytic approach, and context of use, actigraphy may capture walking, limb movement, sedentary behavior, daily routines, and other movement-related aspects of function. Such measures could support early-phase internal decision-making, characterize functional change between clinic visits, and provide supportive evidence alongside established end points.

Recent US Food and Drug Administration (FDA) considerations recognize that digitally derived measures may contribute to multicomponent end points derived from multimodal data [28]. In late-phase ALS trials, validated actigraphy-derived measures could potentially be integrated with the ALSFRS-R or survival through prespecified composite or hierarchical analyses. However, the appropriate statistical approach will depend on the selected measure, its context of use, the relationships among components, and evidence that the combined outcome remains clinically meaningful and interpretable. Specific approaches to composite end point construction should therefore follow the selection and validation of candidate measures.

#### Current Limitations Strengthen the Case for Actigraphy-Derived Measures

Discussions recognized that actigraphy is not without challenges. Candidate features derived from raw actigraphy data, including step count, walking cadence, movement intensity, sedentary time, upper-limb activity, and sleep or rest-activity patterns, remain numerous, and many lack established clinical meaningfulness, defined contexts of use, or regulatory precedent. Interpretation may also be affected by adherence, device placement, analytic pipelines, cost, digital access, and variability in free-living behavior. Actigraphy is also focused in scope: it may capture selected motor domains but will not directly assess all aspects of ALS, including bulbar or respiratory function. Accordingly, the field should prioritize actigraphy-derived measures that are objective, reliable, feasible, low burden, meaningful to people with ALS and clinicians, sensitive to disease progression, targeted to clearly defined functional domains, and suitable for specific contexts of use (Textbox 1). These priorities support the development of focused, repeated, and potentially rapidly responsive measures to supplement current ALS end points, while recognizing that actigraphy-derived measures should be developed as fit-for-purpose tools rather than as substitutes for established end points.

Textbox 1. **Consensus priorities for the current amyotrophic lateral sclerosis (ALS) outcome measures and desired characteristics of supplemental actigraphy-derived measures (source of consensus inputs: consensus priorities summarized were derived from structured stakeholder discussions focused on limitations of current ALS outcome measures and desired attributes of supplemental actigraphy-derived measures).**

**Limitations of existing measures**

- Amyotrophic Lateral Sclerosis Functional Rating Scale–Revised (ALSFRS-R)
  - Subjective
  - Ordinal and noncontinuous
  - Total score may have limited domain-specific interpretability, as similar scores can reflect different patterns of functional impairment
  - May not capture early or subtle functional changes within specific domains
  - Version inconsistencies and administration variability
  - Relatively large sample-size requirements and longer follow-up remain challenges for trials using established end points

- Actigraphy
  - Clinical meaningfulness remains to be established for many candidate measures
  - Current studies often rely on ALSFRS-R or other established clinical outcomes as anchors for validation
  - Proliferation of candidate features has introduced uncertainty around measure selection
  - Cost, digital access, participant/caregiver support, and site/contract research organization readiness may affect implementation
  - Free-living behavior may introduce greater within-person variability than questionnaire-based assessments, requiring appropriate sampling, adherence monitoring, and analytic handling

**What should measures look like?**

- Objective
- Sensitive to disease progression and potential treatment effects
- Reliable, with acceptable signal-to-noise characteristics
- Low burden and acceptable to people with ALS
- Clinically meaningful to patients and clinicians
- Targeted to clearly defined functional constructs or domains
- Capable of capturing relevant domains, such as walking, limb movement, sedentary behavior, or daily routines, depending on the context of use
- Clinically validated and suitable for regulatory engagement
- Potential decision-support value, including prognostic value where supported by evidence
- Applicable across relevant disease stages, while recognizing that specific measures may have domain-specific or stage-specific limitations

### Regulatory Pathways and Staged Adoption of Actigraphy-Derived Measures

#### Regulatory Strategy Should Guide Evidence Generation From Early Development

Perspectives across meetings highlighted the regulatory strategy as a key consideration for the broader adoption of actigraphy in ALS trials. Discussions focused first on the potential use of actigraphy-derived measures in observational studies and early-phase trials, where repeated, objective measures of motor function may support earlier evaluation of functional change. It was also considered how these measures might, over time, be developed for use in late-phase trials. Realizing this broader use will require substantial evidence around device selection, data collection, analytic pipelines, usability, clinical meaningfulness, and context of use. Considering regulatory expectations early can sharpen the field’s approach to evidence generation.

#### Regulatory Pathways Shape How Digitally Derived Measures May Be Adopted

The Clinical Trials Transformation Initiative guide [29], *Regulatory Engagement Opportunities When Developing Digitally Derived Endpoints*, provided context for engaging regulators when developing end points derived from DHTs. Two established routes were considered. First, actigraphy-derived measures may be incorporated within specific drug-development programs, requiring sponsors to justify the measure, context of use, and relevance to treatment evaluation. Second, measures may be developed through drug-development tool pathways, including clinical outcome assessment (COA) qualification, to support broader use within a defined context of use. FDA’s Innovative Science and Technology Approaches for New Drugs program may also be relevant for tools that do not fit existing qualification pathways. Other drug-development tool pathways may be relevant depending on the proposed context of use, although actigraphy-derived measures intended to assess function are likely to align most directly with outcome measure-oriented development.

Examples from adjacent disease areas illustrate both pathways. In product-specific programs, moderate-to-vigorous physical activity has been used as an end point in fibrotic interstitial lung disease (ClinicalTrials.gov ID NCT03267108), and reachable workspace was used as an end point in a phase 3 trial for facioscapulohumeral muscular dystrophy (ClinicalTrials.gov ID NCT05397470). In qualification pathways, a chronic heart failure activity monitor-based end point measure is listed in the FDA COA Qualification Program following acceptance of a letter of intent [30], and stride velocity 95th centile has been qualified by the European Medicines Agency for Duchenne muscular dystrophy studies [31]. Together, these examples show that digitally derived measures can progress through multiple routes, but broad regulatory use requires sustained evidence generation, defined contexts of use, and long development timelines [32].

#### Drug-Specific Development and Broader Qualification Offer Complementary Regulatory Pathways

Actigraphy-derived measures may advance through complementary but operationally distinct pathways. Incorporation within a specific drug-development program may be the more pragmatic route for sponsors seeking to use a novel measure within interventional trials. Measures are often introduced first as exploratory end points, with accumulating evidence supporting progression to more decision-relevant roles. This requires sponsors to justify the measure, the context of use, and the relevance to treatment evaluation within a specific program but may also set a precedent for future studies.

In contrast, COA qualification is independent of a single drug program and supports broader use of a measure across studies within a defined context of use. This pathway may be particularly relevant to consortia, academic groups, patient-centered organizations, or public-private partnerships, seeking standardized tools for broader field use. However, qualification typically requires a larger and more generalizable evidence package and may take longer than program-specific regulatory engagement.

In either pathway, evidence requirements will depend on the context of use and the intended regulatory role of the measure. Recent FDA considerations emphasize that evidence should be commensurate with the intended use and distinguish between the verification of the DHT and analytical and clinical validation of the resulting digitally derived measure [28]. Supporting evidence may include content validity, usability across relevant users and disease stages, test-retest reliability, variability, construct validity against established clinical measures, sensitivity to change, and clinical meaningfulness. For actigraphy in ALS, these requirements reinforce the need to define candidate measures, wear locations, analytical pipelines, patient relevance, and trial contexts before broader regulatory adoption.

#### Accelerated Approval Pathways May Inform Future Actigraphy Development in ALS

Accelerated approval was discussed as a possible future consideration for actigraphy-derived measures in ALS but not as an immediate route to regulatory use. Under FDA-accelerated approval, a drug may be approved for a serious condition using a surrogate end point or intermediate clinical end point that is reasonably likely to predict clinical benefits, with confirmatory evidence required to verify benefits [33]. For actigraphy in ALS, the more relevant concept is likely to be an intermediate clinical end point, rather than a biomarker, if a measure can capture treatment effects on function earlier than established clinical outcomes and predict meaningful clinical benefits. For example, for a lower-limb measure in ambulatory participants, a key milestone would be to prospectively demonstrate that treatment-related preservation of daily walking activity, observed early in a trial, predicts delayed loss of independent ambulation. At present, this remains a longer-term possibility.

Actigraphy-derived measures would first require robust evidence of analytical validity, usability, reliability, sensitivity to change, patient relevance, clinical meaningfulness, and a credible relationship with established measures of benefits such as ALSFRS-R, survival, quality of life, or other patient-relevant functional outcomes. Neurofilament light chain provides an instructive example of how extensive biological and clinical evidence can support regulatory confidence in ALS, including in the context of tofersen [34], but actigraphy-derived measures will require their own evidence base. Accelerated approval should therefore be viewed as a consideration that reinforces early regulatory engagement, not as the primary near-term rationale for actigraphy development.

### Evidence Generation Priorities Increasingly Depend on Harmonization, Fit-for-Purpose Validation, and Adoption-Oriented Implementation

Based on the current state of actigraphy development in ALS, perspectives across meetings highlighted field-wide evidence generation as a central next requirement. Four priorities emerged.

#### Priority 1: Actigraphy-Derived Measures Should Progress Through Staged Adoption

Participants identified a staged framework through which actigraphy-derived measures could gain value and acceptance in ALS trials. Under this framework, drug developers would first use these measures for internal decision-making, particularly in early-phase trials that may be too small or brief to detect robust effects using ALSFRS-R or survival alone. Trials would then use them as supportive measures alongside established outcomes such as ALSFRS-R, survival, quality of life, and patient-reported outcomes. Finally, interventional studies would incorporate them more broadly to build the evidence needed for potential regulatory endorsement in defined contexts of use. For measures to progress across these stages, they will need cumulative evidence of reliability, interpretability, clinical meaningfulness, relevance to people with ALS, and alignment with trial objectives. Table 2 summarizes the evidence priorities across these stages.

**Table 2.** Evidence priorities across staged adoption pathways for actigraphy-derived measures in amyotrophic lateral sclerosis (ALS)^a^.

Adoption pathway	Evidence needed (priority)
Drug developer adoption for internal decision-making	Improved sensitivity or complementarity relative to established end points such as ALSFRS-R^b^; strong measurement properties; feasibility of data collection; participant usability and acceptability; correlations with clinical scales or other relevant anchors; ability to detect disease progression or treatment effect; precedence from other studies; prioritization of a small number of candidate measures; and early regulatory encouragement where appropriate
Supportive use in interventional trials	Strong measurement properties; clearly defined context of use; evidence that the measure captures a functionally relevant construct; patient relevance; usability validation; correlations with clinical scales or other relevant anchors; ability to detect disease progression or treatment effect; prioritization of a small number of candidate measures; and evidence supporting clinical meaningfulness
Broader regulatory acceptance as a study end point	Improved sensitivity or complementarity relative to established end points such as ALSFRS-R; strong measurement properties; explicitly defined measure, device configuration, wear location, and analytic pipeline; clearly defined context of use; evidence of reliability, validity, usability, and clinical meaningfulness; ability to detect treatment effect; prioritization of a small number of candidate measures; and evidence that change in the measure reflects meaningful benefit to people with ALS

a Source of consensus inputs: consensus priorities summarized in this table were derived from stakeholder discussions on the evidence needed to support staged development, adoption, and regulatory advancement of actigraphy-derived measures in ALS. Evidence requirements will vary by context of use, disease stage, trial design, role of the measure, and regulatory pathway.

b ALSFRS-R: Amyotrophic Lateral Sclerosis Functional Rating Scale–Revised.

#### Priority 2: Protocol Harmonization Will Strengthen the Comparability and Interpretability of Evidence

Follow-up discussions emphasized that harmonizing protocols could make evidence more interpretable across studies. Studies should align device selection, wear location, wear duration, sampling frequency, monitoring intervals, compliance tracking, participant support, and data management to improve cross-study comparability and, where appropriate, enable data aggregation. Protocols should specify data-management practices in advance, including data formats, transfer procedures, quality control, missing-data handling, and use of established data standards where applicable.

Current multisite actigraphy studies vary in device platforms, wear locations, sampling rates, wear schedules, and assessment intervals, while also demonstrating areas of convergence (Table 3). Several studies use repeated longitudinal wear periods, sampling rates sufficient for retrospective analysis, and monitoring periods of at least 7 days at monthly to quarterly intervals. These common design features support a best-practice framework that improves comparability while allowing prespecified adaptations for disease phenotype, functional status, participant feasibility, and the intended clinical or trial context. Such a framework could harmonize actigraphy data and accelerate end point validation and broader adoption in ALS.

**Table 3.** Overview of multisite natural history actigraphy studies informing the development of a harmonized digital monitoring framework in amyotrophic lateral sclerosis (ALS).

Study	Country^a^	Population	Status and study size	Device	Wear location	Sampling rate^b^	Wear schedule	Assessment interval
ActiMyo	United States	ALS	Completed; n=30	SYDE (Sysnav)	Wrist and ankle	100 Hz	30 days continuous	Every 3 months
ALS WEAR AI [35]	Canada	ALS	Completed; n=39	ActiGraph GT9X+Fitbit Sense	Wrist (dominant)	30 Hz	8 days	Every 3 months
ARC^c^ Study [14]	United States	ALS+asymptomatic gene carriers+controls	Ongoing; current n=485 ALS; n=30 gene carriers; n=30 controls	ActiGraph GT3X	Both wrists and both ankles	30 Hz	Up to 7 consecutive days per month	Monthly
DIGITIZE (ALL ALS)^d^	United States	ALS+asymptomatic gene carriers+controls	Ongoing; target n=125 ALS; n=25 gene carriers; n=50 controls	Empatica, EmbracePlus, and EmbraceMini	Wrist and ankle (dominant)	Variable (30 Hz minimum)	2 weeks initially, then 1 week per month	Every 4 months
Track ALS	United States	ALS	Completed; n=79	ActiGraph Centrepoint Insight	Wrist (nondominant)	32‐256 Hz	Continuous, over 12 months	Monthly
TRICALS^e^ ORIGIN [18]	Netherlands	ALS	Ongoing; current n=175	ActiGraph GT9X+ActiGraph LEAP	Hip (GT9X) and wrist (LEAP, nondominant)	30 Hz (GT9X hip) and 32 Hz (LEAP wrist)	7 consecutive days per month (≥8 h/d)	Monthly
Remote monitoring in MND^f^	United Kingdom	ALS	Ongoing	GENEActiv	Mid-thigh (nondominant)	100 Hz	7 days continuous	Every 3‐4 months
SMART Wearables [16]	United States	ALS	Completed; n=46	ActiGraph Insight Watch (n=23) and Modus Stepwatch 4 (n=23)	Wrist and ankle (self-selected side)	32 Hz (ActiGraph wrist) and 128 Hz (Modus ankle)	Continuous, up to 6 months	Every 3 months
TechnicALS	Ireland	ALS/PLS^g^	Completed; n=60	ActiGraph GT9X	Wrist (right side) and hip (right side)	32 Hz	7 days per every 4 weeks (8 h/d)	Every 4 weeks
TracMND [15]	Australia	MND (ALS, PLS, PMA^h^) + asymptomatic gene carriers+controls	Ongoing; current n=102 MND; n=97 controls	ActiGraph GT9X+ActiGraph LEAP	Wrist (nondominant); substudy includes both wrists, both ankles, and lower back	30 Hz; 100 Hz for substudy	8 days continuous; substudy at ~2 hours during functional tasks	Every 3‐4 months; substudy once only
Use of ultrasound imaging in MND	United Kingdom	ALS+controls	Ongoing	GENEActiv	Mid-thigh (dominant) and/or wrist (dominant)	100 Hz	7 days continuous	Every 3‐4 months

a Study status and cohort size are shown as reported by site investigators at the time of table compilation.

b All included studies retained raw actigraphy data for retrospective analysis.

c ARC: ALS research collaborative.

d ALL ALS: access for ALL in ALS.

e TRICALS: treatment research initiative to cure ALS.

f MND: motor neuron disease.

g PLS: primary lateral sclerosis.

h PMA: progressive muscular atrophy.

#### Priority 3: Data-Driven Shortlisting Should Prioritize Measures for Cross-Cohort Validation

Although analysis of actigraphy-derived features continues to evolve, the perspectives represented here suggest that the field is ready to prioritize a smaller number of candidate measures for shared development. This does not preclude the continued exploration of new features; rather, it would establish an initial set of analytically defensible measures against which future candidates can be compared. Researchers should use existing and emerging datasets to assess sensitivity to change, reliability, variability, feasibility of data capture, relationships with the ALSFRS-R and other relevant clinical anchors, and patient relevance.

Researchers should also consider sensor location and context of use when prioritizing features. Measures derived from wrist-worn sensors may be suited to assessing upper-limb activity or daily routines, whereas ankle-, thigh-, hip-, or trunk-worn sensors may better capture walking, gait, posture, or mobility-related constructs. Given the heterogeneity of ALS, no single wear location will be equally informative or feasible across all participants or disease stages. Harmonized protocols could therefore retain a core placement for comparability while permitting prespecified alternative or additional placements based on the functional domain of interest, the site and distribution of impairment, ambulatory status, and participant feasibility. Such adaptations should follow prospectively defined decision rules and be incorporated into the analysis rather than determined ad hoc. Researchers should therefore group candidate measures by sensor location, functional domain, analytical approach, and intended trial use, helping sponsors and regulators determine which measures are suitable for specific trial objectives, populations, and disease stages.

Actigraphy-derived measures may also reflect nonmotor influences. Cognitive, behavioral, and neuropsychiatric manifestations, including apathy, depressive symptoms, stereotypies, and compulsive behaviors, could alter free-living activity independently of motor impairment and may also affect adherence. Future studies should characterize relevant nonmotor features, where feasible, and prespecify how they will be considered in participant and adherence support, measure interpretation, and analysis.

#### Priority 4: Adoption-Ready Implementation Requires Early Industry and Regulatory Engagement

Key challenges include regulatory uncertainty, integration into trial workflows, data completeness, participant adherence, site burden, and uncertainty about what constitutes a clinically meaningful actigraphy-derived measure. The field should therefore select candidate measures not only for their statistical performance, including rate of change, variability, and sensitivity to progression, but also for the feasibility of data capture, patient relevance, wear location, data flow, analytic pipelines, and compatibility with interventional trial operations. For actigraphy to gain traction in industry-led trials, protocols must be adoption-ready. Adoption-ready protocols should include a clear rationale for device and wear-location selection, practical participant and caregiver instructions, feasible site workflows, predefined data-management and quality-control procedures, transparent analytic pipelines, and evidence packages aligned with sponsor and regulatory expectations. The field should pursue 3 pathways in parallel: continue collecting and analyzing data to support candidate actigraphy-derived measures; develop best-practice trial methods to simplify adoption; and engage regulators early to clarify expectations for supportive use, qualification, or broader end-point adoption.

### A Perspective on Next Steps for Actigraphy in ALS

Actigraphy has the potential to improve how treatment effects are detected and interpreted in ALS clinical trials by enabling repeated, remote, and objective assessment of movement-related function in daily life. Its value lies not simply in the volume of data collected but in deriving measures that are sensitive to ALS progression, feasible for participants, clinically meaningful, and interpretable within defined contexts of use. To realize this potential, the field must coordinate feasibility testing, harmonize data collection and analysis, validate candidate features, and engage regulators (Figure 4). Four linked priorities emerge.

**Figure 4.**
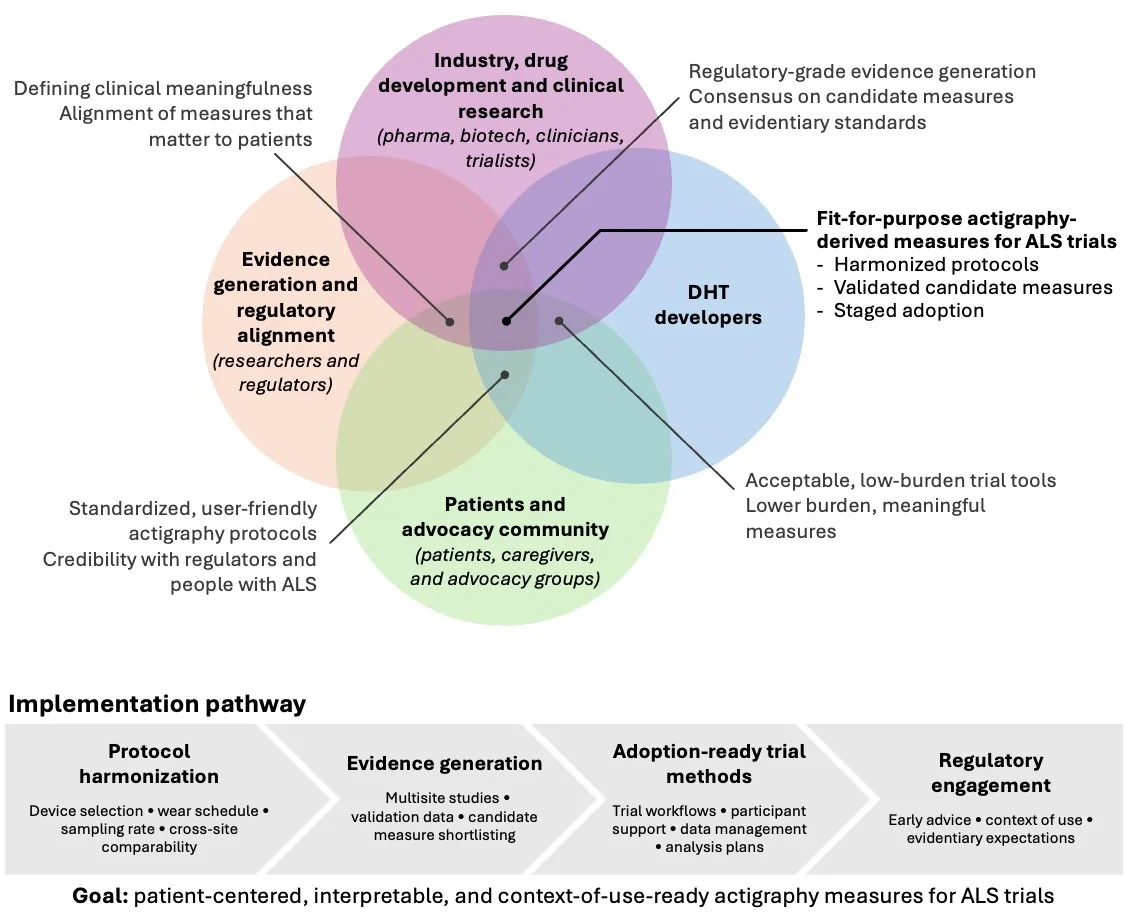
Stakeholder contributions and parallel implementation pathways for actigraphy-derived measures in amyotrophic lateral sclerosis (ALS) trials. Stakeholder groups contribute complementary expertise to the development of fit-for-purpose actigraphy-derived measures, including patient acceptability, trial feasibility, device and protocol usability, evidence generation, and regulatory alignment. Protocol harmonization provides a foundation for evidence generation, adoption-ready trial methods, and early regulatory engagement, supporting development of patient-centered, interpretable, and context-of-use-ready measures for ALS trials. DHT: digital health technology.

First, the field should not view actigraphy-derived measures solely through the lens of formal end point qualification. Rather, it should develop them as fit-for-purpose tools that generate value across observational studies, early-phase trials, sponsor decision-making, supportive end point use, and, potentially, future regulatory submissions. The field will likely progress in stages, first using candidate measures to understand disease progression and support exploratory analyses before adopting them more broadly in interventional trials.

Second, evidence generation, protocol harmonization, and feature selection are closely linked. Studies should incorporate best-practice approaches to device platforms, wear locations, monitoring schedules, adherence monitoring, data flows, feature selection, and analytical pipelines within validation itself to support cross-study comparability, shared evidence generation, and interpretable evaluation of candidate measures. The field should prioritize candidate measures according to their sensitivity to ALS progression relative to variability, feasibility, clinical meaningfulness, patient relevance, and alignment with the context of use.

Third, patient-centeredness must remain central. Survey findings and stakeholder perspectives indicate that people with ALS are open to actigraphy when the purpose is clear, participant burden is minimized, and the data generated are likely to improve care or trial participation. People with lived experience should remain involved to ensure that selected measures capture outcomes that matter in daily life and remain feasible across disease stages and motor phenotypes.

Finally, the field should pursue 4 activities in parallel: collect and analyze data to select promising features, develop best-practice trial methods to simplify adoption, build evidence packages aligned with sponsor and regulatory expectations, and maintain engagement with regulators. By collaborating to develop harmonized, patient-centered, and fit-for-purpose actigraphy methods, stakeholders may strengthen ALS trial design and support more efficient evaluation of emerging therapies.

## Conclusions

Taken together, this viewpoint calls on the field to shift its emphasis. The field should not simply advocate for actigraphy in principle; it should build a shared framework to collect and harmonize data, analyze and select features, align with regulatory expectations, and incorporate actigraphy into ALS clinical trials. For people with ALS, the value of this work is practical: more sensitive and participant-friendly tools, better aligned with lived experience and trial needs, may improve the efficiency and interpretability of ALS trials, and support more effective evaluation of emerging therapies.

## Abbreviations

ALS: amyotrophic lateral sclerosis
ALSFRS-R: Amyotrophic Lateral Sclerosis Functional Rating Scale—Revised
COA: clinical outcome assessment
DHT: digital health technology
FDA: US Food and Drug Administration
